# Computational model of pesticide deposition distribution on canopies for air-assisted spraying

**DOI:** 10.3389/fpls.2023.1153904

**Published:** 2023-05-08

**Authors:** Hanjie Dou, Qi Li, Changyuan Zhai, Shuo Yang, Chunjiang Zhao, Yuanyuan Gao, Yakai He

**Affiliations:** ^1^ National Engineering Research Center for Information Technology in Agriculture, Beijing, China; ^2^ College of Agricultural Engineering, Jiangsu University, Zhenjiang, China; ^3^ Intelligent Equipment Research Center, Beijing Academy of Agriculture and Forestry Sciences, Beijing, China; ^4^ Institute of New Materials Technology & Equipment, Chinese Academy of Agricultural Mechanization Sciences Group Co., Ltd, Beijing, China

**Keywords:** crop protection, air-assisted spraying, canopy, pesticide deposition, computational model

## Abstract

The deposited pesticide distribution in fruit tree canopies is crucial for evaluating the efficacy of air-assisted spraying in orchards. Most studies have determined the impact of pesticide application on pesticide deposition on canopies without a quantitative computational model. In this study, an air-assisted orchard sprayer with airflow control was used to perform spraying experiments on artificial and peach trees. In the spraying experiment on an artificial tree, a canopy with leaf areas ranging from 2.54~5.08 m^2^ was found to require an effective air speed of 18.12~37.05 m/s. The canopy leaf area, air speed at the sprayer fan outlet and spray distance were used as test factors in a three-factor five-level quadratic general rotational orthogonal test to develop a computational model for pesticide deposition at the inner, outer and middle regions of a fruit tree canopy with *R* ^2^ values of 0.9042, 0.8575 and 0.8199, respectively. A significance analysis was used to rank the influencing factors for the deposited pesticide distribution in decreasing order of significance as follows: the spray distance, leaf area and air speed for the inner region of the canopy, followed by the spray distance, air speed and leaf area for the middle and outer regions of the canopy. The results of the verification test conducted in a peach orchard showed that the computational errors of the pesticide deposition model for the inner, middle and outer regions of the canopy were 32.62%, 22.38% and 23.26%, respectively. The results provide support for evaluating the efficacy of an air-assisted orchard sprayer and optimizing the sprayer parameters.

## 1 Introduction

Chemical pesticides play a dominant role in pest control for fruit trees. Pesticides are applied on fruit trees approximately 8-15 times a year, which contributes approximately 30% to the total workload (Van de Zande et al., 2008; Dekeyser et al., 2014). Air-assisted sprayers are widely used for orchard protection because using an airflow to transport droplets enhances pesticide penetration and adhesion to leaves (He, 2020; Zheng et al., 2020). The use of a high air speed for air-assisted spray application induces pesticide drift, whereas using an airflow that is too small to penetrate the canopy affects the efficacy of pest control (Zhai et al., 2018). Pesticide deposition on fruit tree canopies is key for evaluating the efficacy of air-assisted sprayers. Establishing a rule and a computational model for pesticide deposition on a canopy are very important for improving the efficacy of orchard air-assisted sprayers and optimizing the sprayer operation parameters (Teske et al., 2011).

Scholars in China and around the world have carried out many studies to determine how the deposited pesticide distribution in fruit tree canopies is affected by the pesticide application operation parameters (the spraying speed, fan speed, spray distance, spray pressure, nozzle flow rate, etc.). Jadav et al. (2019) studied the impact of different operation parameters for pesticide spraying on the deposited pesticide distribution in canopies. The spraying speed was found to significantly affect the deposited pesticide distribution. Jiang et al. (2016) combined air-assisted pesticide application and Internet of Things (IoT) technologies to perform a comparative test on sprayers with and without an air-assisted spraying function. Higher pesticide deposition was found using air-assisted spraying than without air-assisted spraying and saved over 30% of the pesticide used. Qiu et al. (2016) studied the impact of different fan speeds on pesticide deposition for pear trees. Fan speed was found to significantly affect pesticide deposition, although fan speeds exceeding 1,300 r/min reduced the deposition rate and coverage. Gu et al. (2020) studied the influence of spraying parameters, such as the fan speed and spray distance, on the deposited pesticide distribution in a kiwi fruit orchard using the orthogonal test method and established a regression equation to optimize and verify the parameters. Ding et al. (2020) studied the impact of spraying parameters, such as air speed, on pesticide deposition to provide a data reference for field spraying operations. Hong et al. (2018) conducted a computational fluid dynamics (CFD) simulation on the airflow inside a canopy and found that air speeds above a well-defined range reduced the quantity of pesticide deposited on the canopy. Duga et al. (2015) reported that the airflow distribution of a sprayer can affect pesticide deposition in the vertical section of the canopy and that canopy characteristics, such as leaf area and volume, significantly impact pesticide deposition. Sun and Liu (2019) studied a variety of fruit trees to establish a mathematical model for the second exponential of the droplet penetration ratio based on the leaf area density, canopy sampling depth and air speed. The sampling depth was found to have the most significant impact on droplet penetration into the canopy. Zhu et al. (2022) and Zhai et al. (2021) employed a porous media model, and Duga et al. (2017) and Zhang et al. (2022) used a simplified equivalent porous media model to study the influence of canopy shape and leaf area density on the airflow field. The complex process of transporting droplets by airflow was simulated, and the effect of the airflow on the pesticide droplet distribution in the canopy was determined.

Studies have shown that the effective deposition of droplets inside the canopy can be improved by changing the spraying parameters according to the characteristics of the fruit tree canopy. The deposition of droplets involves the complex motion of trajectory spreading on the surface of branches and leaves through the canopy gap. It is difficult to determine the penetration law and pesticide deposition distribution characteristics for different areas of a target canopy (Endalew et al., 2010; Otto et al., 2018; Liu et al., 2021). A current challenging research problem is how to quantify pesticide deposition on different canopy areas based on the droplet deposition law for the canopy. Li et al. (2020) studied the impact of the spray distance, air speed at the air outlet of the sprayer fan and droplet size on pesticide deposition on the leaf surface and established a prediction model for the deposition state of droplets on the leaf surface. Farooq et al. (2001) established a simulation model to predict pesticide deposition on a fruit tree canopy. Shani (2020) used dimensional analysis to evaluate the influence of spraying parameters on the weight of pesticides deposited on a canopy and established a mathematical model to predict the weight of deposited pesticides. Shani (2021) subsequently analyzed the relationship between the spraying operation parameters and pesticide deposition for a fruit tree canopy and established a computational model for pesticide deposition under different operating conditions. This model was theoretically derived, and its applicability must be verified by orchard tests.

The objective of this study was to establish a computational model for pesticide deposition on different canopy areas considering the main influencing factors for pesticide deposition (canopy leaf area, air speed at the air outlet of the fan and spray distance). The impact of the main influencing factors on pesticide deposition in the inner, middle and outer regions of the canopy was determined. Thus, the results provide support for evaluating the efficacy of an orchard air-assisted sprayer and optimizing the sprayer performance parameters.

## 2 Materials and methods

### 2.1 Orchard air-assisted sprayer with airflow control

**Figure 1:**
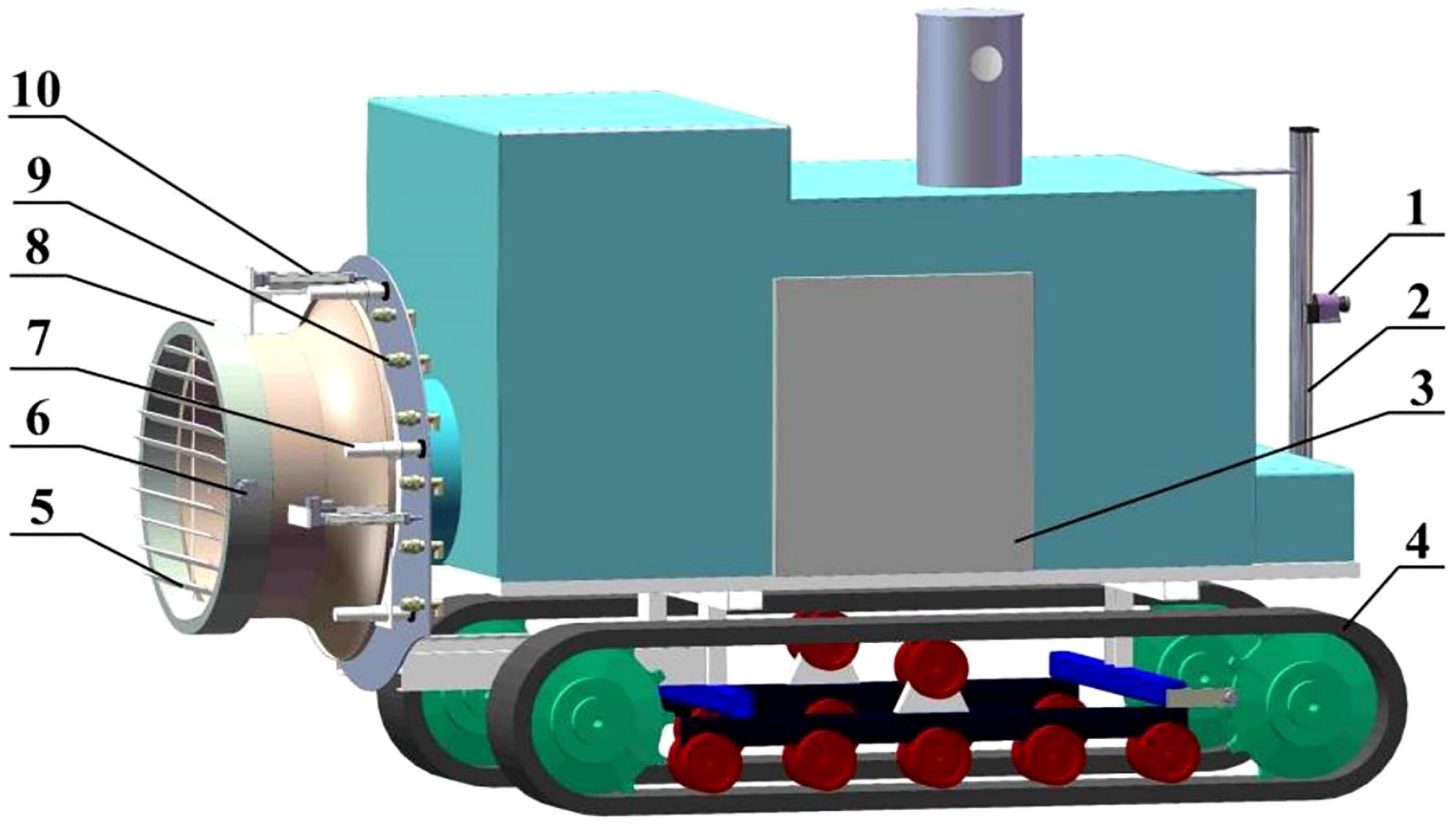
Structure chart for the complete crawler-type air-assisted sprayer. 1. LiDAR 2. Screw rod module 3. Control system 4. Crawler base 5. Louvers 6. Stepping motor 7. Slide rail 8. Fan 9. Nozzle 10. Electric drive pusher.

**Figure 1** shows the orchard air-assisted sprayer with airflow control that was used to perform tests in this study. The fan speed and areas of the air inlet and outlet of the sprayer could be independently regulated. The main components of the sprayer are a crawler base, a control system, light detection and ranging system (LiDAR), a nozzle, a fan, louvers and slide rails. LiDAR obtains information on the fruit tree canopy characteristics (the position, canopy volume, leaf area, etc.) in real time. The fan is driven by an AC motor, and its speed can be adjusted in the range of 0–2923 r/min. The louvers are installed at the air inlet through an expansion cylinder, and the area of the air inlet can be adjusted by controlling the angle of the louvers using a stepping motor. The fan cylinder is connected to the back panel through an electric drive pusher, which can be moved to adjust the area and thereby the opening of the air outlet.

**Figure 2:**
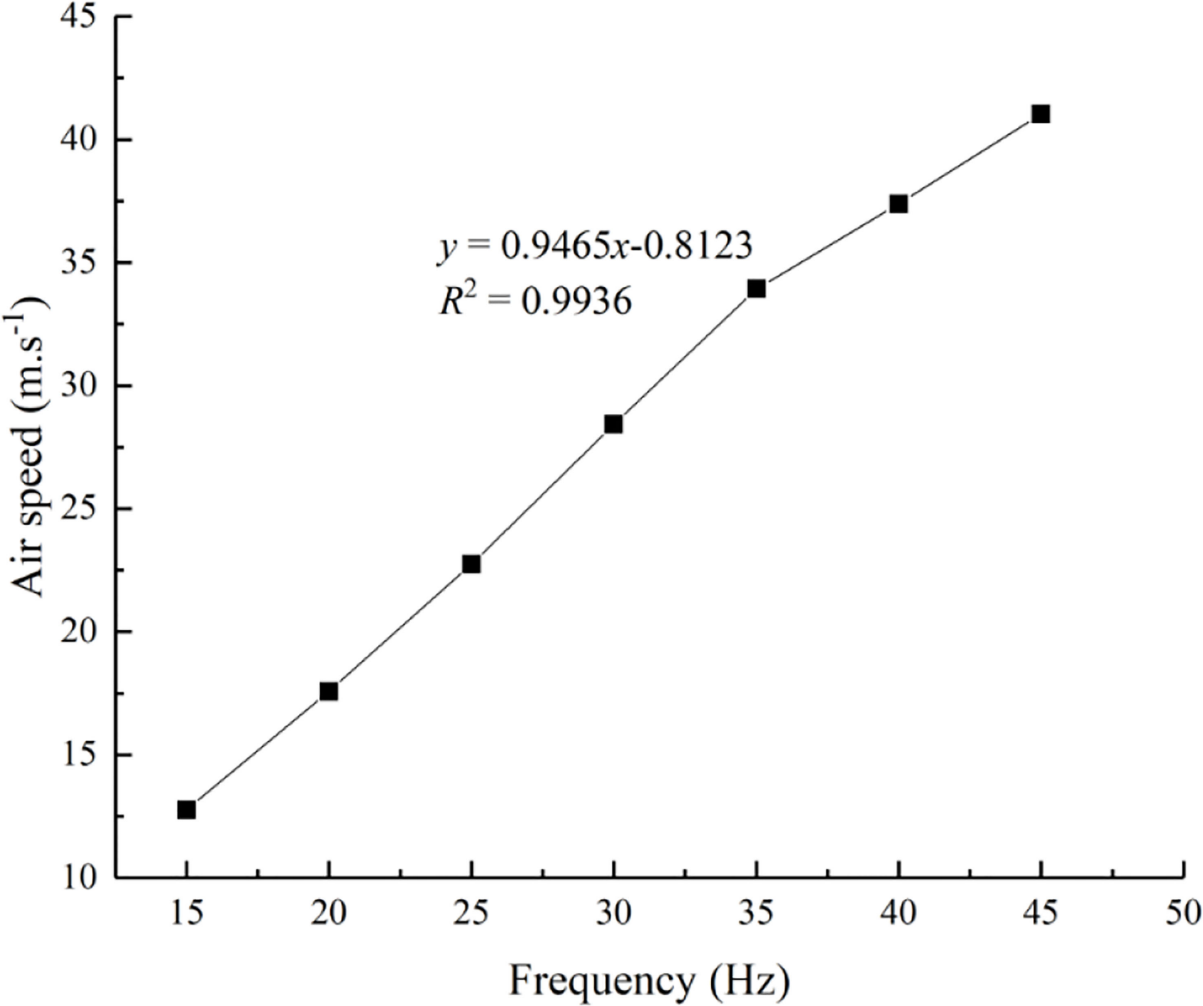
Relationship between the inverter frequency and the air speed at the fan outlet. In the equation, *x* represents the inverter frequency, Hz, and *y* denotes the air speed at the air outlet of the fan, m/s.

The effect of the air speed at the fan outlet on the deposition and distribution laws of droplets in different regions of a fruit tree canopy was determined. Air speed was regulated by adjusting the frequency of the fan’s drive motor inverter. Because the nozzles at the fan outlet were positioned at equal intervals, one air speed measurement point was set at each nozzle position of the fan outlet. A soft blue ribbon of a certain length was tied to each nozzle position to determine the airflow direction at that position, which was used to document the direction of the air speed sensor (8455-300, TSI Company, USA) to rapidly measure the air speed at each nozzle position. The average value of air speed of each nozzle position was taken as the air speed at the fan outlet. The relationship between the inverter frequency and the air speed at the fan outlet was shown in **Figure 2**. There was a good linear relationship between the inverter frequency and the air speed, which was used to calculate the air speed at the fan outlet under different inverter frequencies.

### 2.2 Artificial tree canopy

**Figure 3:**
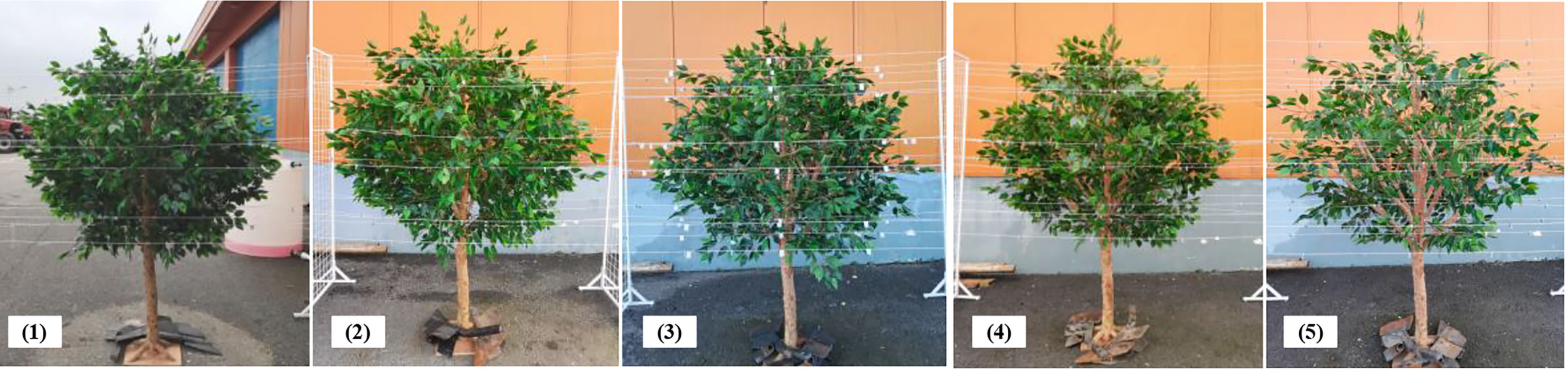
An artificial canopy with different leaf areas. (1) leaf area = 5.08 m^2^, (2) leaf area = 4.57 m^2^, (3) leaf area = 3.81 m^2^, (4) leaf area = 3.05 m^2^, (5) leaf area = 2.54 m^2^.

An artificial tree canopy was used to simulate changes in fruit tree canopies in different growth periods. The artificial tree had a height of 2.0 m, a crown width of 1.6 m and a canopy height of 1.2 m. The canopy density was changed by manually picking and attaching leaves based on leaf changes of peach canopy at different growth stages obtained by pre-experiment. The artificial tree consisted of 4583 large leaves and 913 small leaves, based on leaf statistics. An instrument for measuring the leaf area (Shandong Fangke Instrument Co., Ltd., YMJ-G) was used to scan 10 groups of leaves. The average leaf areas for large and small leaves were 19.21 cm^2^ and 14.79 cm^2^, respectively. Specific numbers of leaves were then picked and arranged to create a canopy with different leaf areas, as shown in **Figure 3**.

### 2.3 Tests to determine the required air speed range for the artificial tree canopy

**Figure 4:**
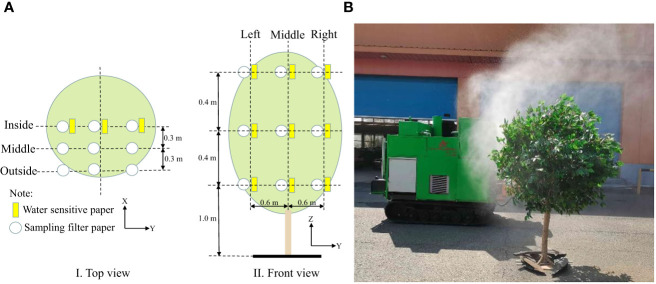
Layout of sampling points and test. **(A)** Sampling layout. **(B)** Spraying test. X = spray direction, Y = sprayer travel direction, and Z = tree height.

The range of required air speed for air-assisted spraying on canopies with different leaf areas was determined using spraying tests that were designed according to the national standards of China, i.e., GB/T 3244-2015 Crop Protection Equipment - Field Measurement of Spray Distribution in Tree and Bush Crops (Yan et al., 2015). The tests were carried out by placing water-sensitive papers (size: 2.5 × 5 cm) on the front and back sides of the leaf along the plane of the tree trunk center on the sprayed side of the artificial tree canopy to evaluate droplet deposition under different air speeds. The layout of the papers is shown in **Figure 4A**. During the test, the spray pressure was set to 1.0 MPa, and the inverter frequency was adjusted to set the air speed to 12.76 m/s, 17.58 m/s, 22.74 m/s, 28.42 m/s, 33.93 m/s, 37.38 m/s and 41.03 m/s. A remote-controlled sprayer was used to spray a solution at 1.0 m/s along the spray center 3.0 m from the tree trunk. After the droplets on the water-sensitive papers dried, the papers were placed in a bag, which was labelled according to the number of papers and taken to the laboratory for analysis.

### 2.4 Tests for droplet deposition on an artificial-tree canopy

The following test factors were used: leaf area, air speed for fan outlet and spray distance. The quantities of pesticide deposited at the inner, middle and outer positions of the artificial tree canopy were used as the response values. A three-factor five-level quadratic general rotational orthogonal test was designed using Design-Expert 8.06 software (Xu and He, 2010). **Table 1** shows the factor coding. The canopy leaf area and the air speed were determined using the test procedure described in Sections 2.2 and 2.3. The planting pattern of orchards in China was used to determine the spray distance from the tree trunk to the sprayer center as 1.5 to 3.0 m, and the value of γ was 1.682. The γ stands for asterisk arm.

**Table 1:** Factor coding table.

Factor level	Leaf area (m^2^)	Air speed (m·s^-1^)	Spray distance (m)
Zero level (z_0_)	3.81	27.59	2.25
Radius variation (△)	0.76	5.62	0.45
-γ	2.54	18.12	1.50
-1	3.05	21.96	1.80
0	3.81	27.59	2.25
1	4.57	33.21	2.70
γ	5.08	37.05	3.00

The test was conducted at the National Precision Agriculture Research and Demonstration Base in Xiaotangshan, Changping District, Beijing, China. The droplet pesticide depositions for different test combinations were obtained by arranging filter papers for sampling (9 cm in diameter, Special Paper Co., Ltd., Hangzhou, China) at different positions in the fruit tree canopy. The layout of the filter papers is shown in **Figure 4A**.

**Figure 5:**
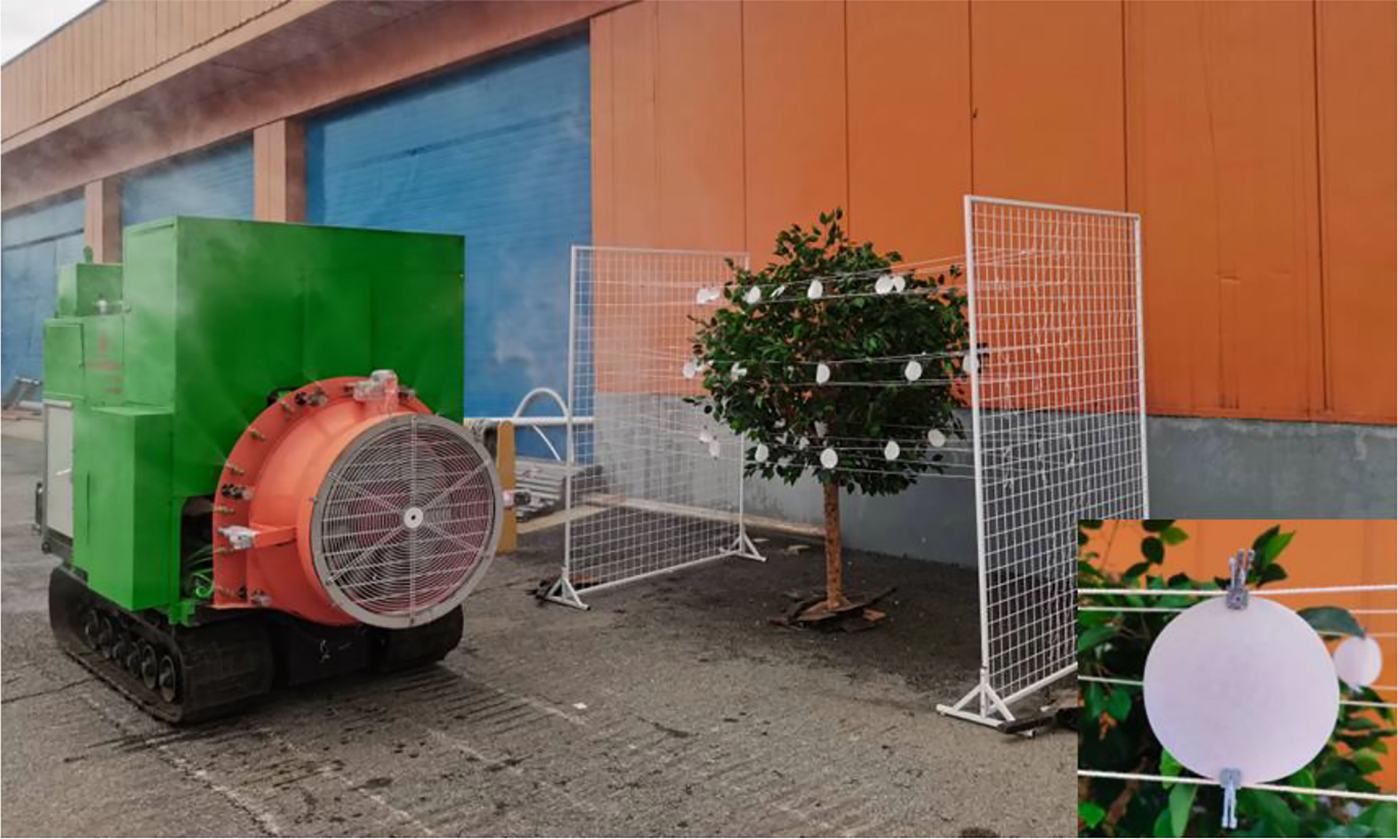
Pesticide deposition test for a fruit tree canopy.

A tracer (rhodamine B, Tianjin Kemiou Chemical Reagent Co., Ltd.) was used instead of a pesticide in the test. The spray pressure was set to 1.0 MPa. We used the specifications in **Table 1** to regulate the air speed, vary the leaf area of the artificial tree canopy, and remotely control the distance of the sprayer to achieve a spray velocity of 1.0 m/s, as shown in **Figure 5**. During the test, a self-developed small field weather station was used to monitor the ambient temperature, humidity, wind velocity and wind direction in real time. For a southeast wind, the average ambient temperature, relative humidity and wind velocity were 21.71°C, 45.95% and 0.70 m/s, respectively. The test results showed that a small quantity of pesticide was deposited on the nonsprayed side of the fruit tree canopy. Deposition on the nonsprayed side of the canopy was neglected in calculating the quantity of pesticide deposited on different regions of the sprayed canopy side.

### 2.5 Orchard tests

The established computational model for pesticide deposition on a canopy was validated by conducting a spraying test in a peach orchard at the National Precision Agriculture Research and Demonstration Base in Xiaotangshan, Changping District, Beijing, China. The test was performed on 5-year-old peach trees, Ruiguang No. 8, with a row spacing of 4.5 m and a tree spacing of 5.0 m. Point cloud data for the canopy were obtained with LiDAR placed in front of the sprayer. The canopy volume was determined to be 5.39 m^3^ using a detection method that was previously developed by the research team (Gu et al., 2021). The method used to calculate the canopy leaf area of the artificial tree was used to compile statistics to determine the number of leaves in the fruit tree canopy. A preliminary test was carried out to statistically analyze canopy leaf changes, and the statistical results show that the area of a single leaf was divided by 25 cm^2^ into large leaves and small leaves, and the distribution proportion of the two types of leaves in the canopy was close to the same, which was used to classify large leaves and small leaves in orchard test. We randomly picked leaves and used the statistical method to determine the leaves areas. We scanned the leaves with the instrument for measuring the leaf area and determined the average areas of the leaves to be 36.44 cm^2^ and 21.59 cm^2^ for large leaves and small leaves, and the number of large leaves and small leaves were 914 and 782, respectively. The canopy leaf area was 5.02 m^2^. We used the test results for the artificial tree canopy to design a spraying test for the orchard. The test combinations are shown in **Table 2**.

**Table 2:** Parameters used for the orchard spraying test.

Test No.	Leaf area (m^2^)	Air speed (m·s^-1^)	Spray distance (m)
1	5.02	38.94	3.00
2		35.15	3.00
3		33.26	2.70
4		37.05	2.70
5		32.62	2.25

**Figure 6:**
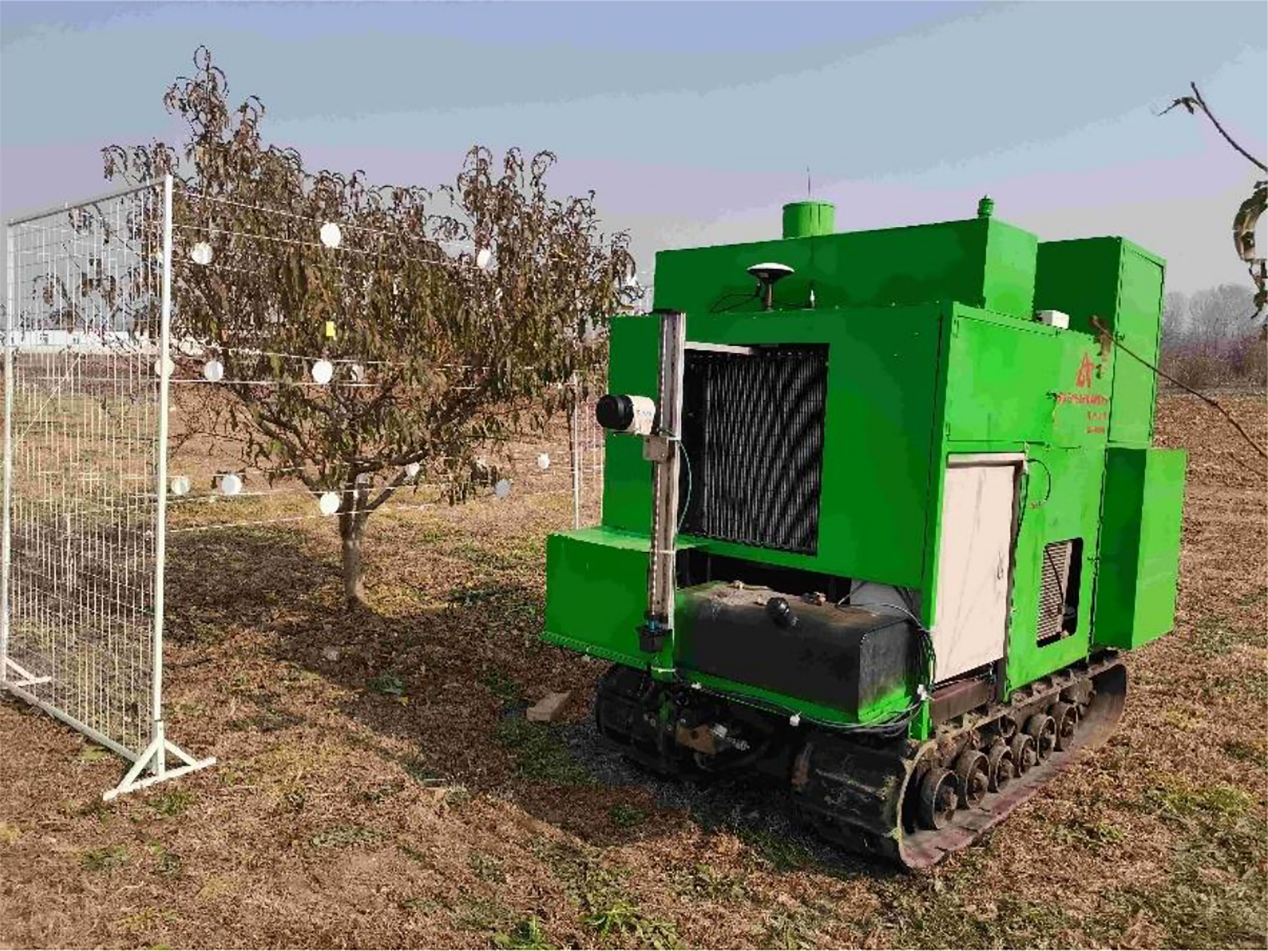
Orchard spraying test.

The method described in Section 2.4 was used to arrange filter papers for sampling the peach tree canopy, as shown in **Figure 6**. A rhodamine tracer was used instead of a pesticide. The spray pressure was set to 1.0 MPa. The fan speed was set according to the air speed values given in **Table 2**. We drove the sprayer to achieve a 1.0 m/s spray from east to west and collected the filter papers in a marked opaque white plastic box. Upon completion of the test, the collected filter papers were taken to the laboratory for data analysis. For a southeast wind, the average ambient temperature, relative humidity and wind velocity were 20.85°C, 48.03% and 0.74 m/s, respectively.

### 2.6 Test data analysis

**Figure 7:**
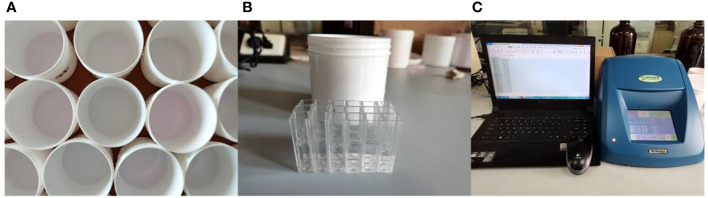
Measurement of the rhodamine solution content on the filter papers used for sampling. **(A)** Collection container. **(B)** Cuvette. **(C)** Fluorometer.

The water-sensitive papers were scanned using a TSN450 scanner developed by ShenZhen Tiancai Electronic Co., Ltd. to obtain greyscale images. These images were analyzed using droplet deposition analysis software developed by Chongqing Liuliu Shanxia Co., Ltd. to determine changes in the droplet coverage and deposition point density at different air speeds. A fluorometer (Turner Designs, Inc., San Jose, Cal) was employed to measure the content of the rhodamine solution on the filter papers used for sampling, as shown in **Figure 7**. Each filter paper was placed in a beaker, and distilled water was added to the beaker up to a volume of 80 mL. The filter paper was allowed to soak in the water for 10 min and removed from the beaker. A portion of the solution was transferred to a cuvette, which was placed in a fluorometer to measure the content of the rhodamine solution. Each sample was measured three times, and the average value is reported as the final measured value.

The measured content of the rhodamine solution on the filter paper was used in Equation (1) to calculate the quantity of pesticide deposited per unit area of the filter paper at different positions in the canopy (Dou et al., 2021).


(1) $$Deposition=\frac{C_{paper}\times V}{C_{tank}\times S_{paper}\times R}$$

where *Deposition* is the quantity of the tracer agent deposited on the filter paper, mL/m^2^; *C*_paper_ is the concentration of the rhodamine solution on the filter paper, mg/mL; *V* is the volume of distilled water used for washing, mL; *C*_tank_ is the concentration of the mother solution, mg/mL; *S*_paper_ is the area of the filter paper, m^2^; and *R* is the recovery rate of the solution, which was measured to be 87.29%.

The single filter paper at each sampling point covered a circular area with a diameter of 9 cm. To calculate the spray deposition quantity at different canopy regions, a rectangular area surrounding each filter paper was outlined. The quantities of spray deposition in the rectangular area were the product of the quantities of pesticide deposition on each filter paper and the area of the rectangle, and the total quantities of spray deposition in all the rectangles was considered the quantities of pesticide deposited at current canopy area. The quantities of pesticide deposited at the inner, middle and outer positions of the canopy was calculated using Equation (2).


(2) $$Deposition_{Canopy}=\frac{\sum_{i=1,j=1}Deposition_{ij}c_{ij}}{S_{\text{i}}}$$

Where *Deposition*_Canopy_ is the quantities of pesticide deposited at the inner, middle and outer positions of the canopy, mL/m^2^; *Deposition*_ij_ is the quantity of the tracer agent deposited on the filter paper for the *j* sampling point in the *i* region, mL/m^2^; *c*_ij_ is the area of the rectangle for the *j* sampling point in the *i* region, m^2^; *S*_i_ is the canopy section area, m^2^; *i* is 1, 2 and 3 for the inner, middle and outer positions of the canopy, respectively; *j* is the number of sampling points at different canopy regions.

## 3 Test results and analysis

### 3.1 Tests to determine the required air speed range for the artificial tree canopy

**Figure 8:**
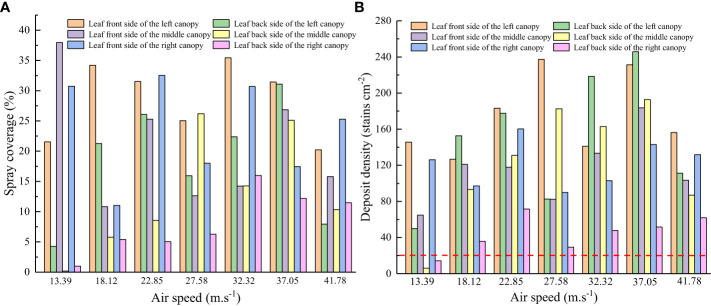
Changes in spray coverage and deposit density for different air speeds. **(A)** Spray coverage. **(B)** Deposit density.

The data obtained using the water-sensitive paper were used to determine changes in droplet coverage and deposition point density for different air speeds, as shown in **Figure 8**.

**Figure 8** shows that the spray coverage on the front and back sides of the canopy leaf changes as the air speed increases. At an air speed of 13.39 m/s, there is low coverage on the back of the leaf, and the deposit density is less than 20 deposits/cm^2^. The number of droplets deposited on a crop must exceed 20 deposits/cm^2^ during the spraying process for effective pest control (Salcedo et al., 2020). Therefore, the air speed should be maintained above 13.39 m/s during the testing process. By comparison, at an air speed of 37.05 m/s, there is comparable coverage on the front and back sides of a tree leaf, and the mist spray is uniform. Increasing the air speed further results in a significant difference in droplet coverage on the front and back sides of the leaf and deteriorates the uniformity of the mist spray, because the droplet size increases with the air speed. However, the adhesiveness of a droplet to the leaf surface decreases beyond a well-defined range of drop sizes. In summary, an air speed range of 18.12–37.05 m/s is required for canopy leaf areas between 2.54 m^2^ and 5.08 m^2^.

### 3.2 Test for droplet deposition on an artificial tree canopy

#### 3.2.1 Computational model for pesticide deposition in different canopy regions

We used the analysis method for the test data described in Section 2.6 to calculate the quantity of pesticide deposited on different canopy regions for different test combinations. The results are shown in **Table 3**.

**Table 3:** Test data for pesticide deposition on a canopy for different test combinations.

Test No.	Factors			Responses		
	*A* (m^2^)	*B* (m·s^-1^)	*C* (m)	*Y*_1_ (mL.m^-2^)	*Y*_2_ (mL·m^-2^)	*Y*_3_ (mL·m^-2^)
1	4.57	33.21	2.70	6.80	9.96	9.67
2	4.57	33.21	1.80	9.56	18.75	20.89
3	4.57	21.96	2.70	5.48	11.91	15.65
5	3.05	33.21	2.70	9.18	10.37	17.17
6	3.05	33.21	1.80	7.29	14.25	17.69
7	3.05	21.96	2.70	6.91	10.60	16.96
8	3.05	21.96	1.80	12.98	21.10	26.12
9	2.54	27.59	2.25	9.16	10.75	18.83
10	5.08	27.59	2.25	7.95	11.33	14.82
11	3.81	18.12	2.25	7.54	20.29	22.27
12	3.81	37.05	2.25	8.94	13.41	16.85
13	3.81	27.59	1.50	13.36	18.86	23.97
14	3.81	27.59	3.00	8.38	12.70	15.33
15	3.81	27.59	2.25	8.83	13.71	17.55
16	3.81	27.59	2.25	10.02	13.90	18.18
17	3.81	27.59	2.25	10.74	16.16	19.97
18	3.81	27.59	2.25	11.39	16.75	19.19
19	3.81	27.59	2.25	12.91	16.72	21.27
20	3.81	27.59	2.25	9.36	13.04	18.14

In the table, A represents the canopy leaf area; B represents the air speed; C represents the spray distance; and Y_1_, Y_2_ and Y_3_ represent the quantities of pesticide deposited at the inner, middle and outer positions of the canopy, respectively.

Design-Expert 8.06 software was used to determine the regression equation, regression coefficient and lack-of-fit for the regression model. The variance analysis results are shown in **Table 4**. The overall *P* values of the computational model for the inner, middle and outer regions of the canopy are 0.0092, 0.0032 and 0.0005, respectively, which are less than 0.05, indicating that the regression models relating the test factors (*A*, *B* and *C*) to the response variables (*Y*_1_, *Y*_2_ and *Y*_3_) are significant. The *P* values for the lack-of-fit of the model for the inner, middle and outer regions of the canopy are 0.7401, 0.2943 and 0.2065, respectively, which are all greater than 0.05, indicating that the lack-of-fit values are not significant, the lack-of-fit error between the model equation and the fit to the data is small, and the regression model effectively fits the experimental data. The signal-to-noise ratios (SNRs) for the measurements of the model accuracy for the inner, middle and outer regions of the canopy are 9.354, 7.917 and 12.907, respectively. These ratios are all greater than 4, indicating that the model has high reliability. A regression analysis was used to obtain Equations (3)-(5) for the quantities of spray deposited per unit area (mL/m^2^) in the inner, middle and outer regions of the canopy (denoted by *Desposition_Inner_*, *Desposition_Middle_* and *Desposition_Outer_*, respectively) in terms of the canopy leaf area (*A*, m^2^), air speed (*B*, m/s) and spray distance (*C*, m). The *R* ^2^ values of Equations (3), (4) and (5) are 0.8199, 0.8575 and 0.9042, respectively.

**Table 4:** Variance analysis for the regression model.

Sources	Pesticide deposition (mL·m^-2^)									
	Inner			Middle			Outer			
	Degree of freedom	F value	P -values Prob>F	Degree of freedom	F value	P -values Prob>F	Degree of freedom	F value	P -values Prob>F	
Model	9	5.06	0.0092	9	6.69	0.0032	9	10.48	0.0005	significant
*A*	1	0.88	0.3701	1	0.86	0.3751	1	2.75	0.1284	
*B*	1	0.21	0.6550	1	10.50	0.0089	1	21.82	0.0009	
*C*	1	20.74	0.0011	1	36.03	0.0001	1	54.15	<0.0001	
*AB*	1	0.38	0.5527	1	0.21	0.6567	1	0.88	0.3698	
*AC*	1	1.96	0.1914	1	0.50	0.4972	1	7.62	0.0201	
*BC*	1	10.24	0.0095	1	1.77	0.2130	1	3.72	0.0826	
*A^2^*	1	5.13	0.0469	1	7.11	0.0236	1	2.41	0.1515	
*B^2^*	1	6.74	0.0267	1	1.83	0.2055	1	0.29	0.5991	
*C^2^*	1	0.013	0.9102	1	0.38	0.5531	1	0.38	0.5522	
Lack of fit	5	0.54	0.7401	5	1.67	0.2943	5	2.18	0.2065	not significant
SNRs		9.354			7.917			12.907		


(3) $$\begin{array}{c} Deposition_{Inner}=7.73+12.36A-0.04B-13.26C+0.07A\cdot B-1.90A\cdot C+\\ 0.58B\cdot C-1.36A^{2}-0.03B^{2}+0.20C^{2} \end{array}$$

(4) $$\begin{array}{c} Deposition_{Middle}=44.49+19.98A-2.60B-18.49C+0.07A\cdot B-1.42A\cdot C+\\ 0.36B\cdot C-2.39A^{2}+0.02B^{2}+1.55C^{2} \end{array}$$

(5) $$\begin{array}{c} Deposition_{Outer}=18.04+23.60A-1.37B-7.92C-0.14AB-5.03AC+\\ 0.47BC-1.26A^{2}+7.91\times 10^{-3}B^{2}+1.40C^{2} \end{array}$$

#### 3.2.2 Analysis of normal plot of residuals

**Figure 9:**
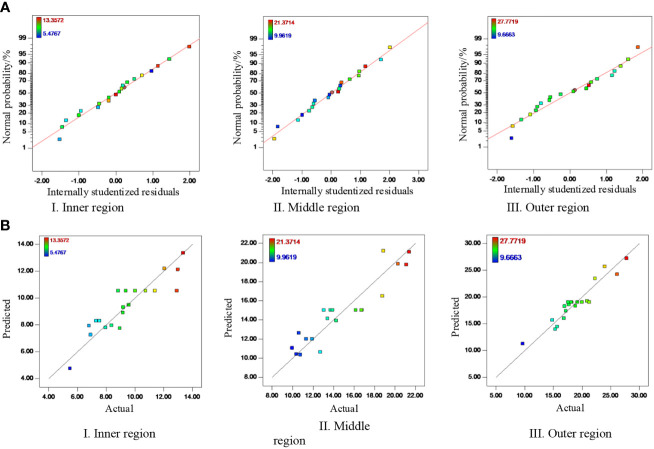
Analysis of a normal plot of the model residuals. **(A)** Normal plot of residuals. **(B)** Corresponding relation between the values predicted by the regression model and the actual values.

The accuracy of the regression model was further analyzed by using Design-Expert 8.06 to generate a normal plot of residuals for the regression model and the corresponding relation between the values predicted by the regression model and the actual values. The results are shown in **Figure 9**. **Figure 9A** shows that 95% of the residuals are distributed within the standard range around a straight line, indicating a normal error distribution. **Figure 9B** shows that the model predictions and actual values are consistent and follow good linear distributions. In summary, the normal distribution of the regression model and the standard prediction of errors can be used to calculate the quantity of pesticide deposited on the fruit-free canopy.

#### 3.2.3 Response surface analysis

**Figure 10:**
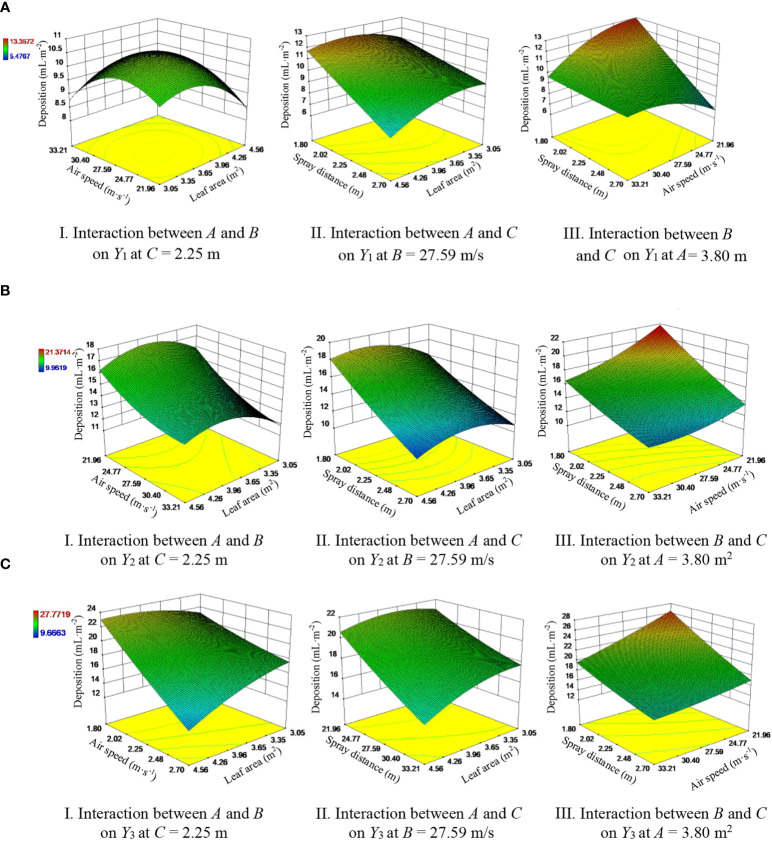
Response surface showing the influence of factor interactions on pesticide deposition on the inner, middle and outer regions of the canopy. **(A)** Inner region of the canopy. **(B)** Middle region of the canopy. **(C)** Outer region of the canopy.

**Figure 10** shows the response surface for the regression equation to analyze the influence of the interaction of any two factors of *A*, *B* and *C* on *Y*_1_, *Y*_2_ and *Y*_3_.

**Figure 10.A.I** shows that for *C* = 2.25 m, increasing *A* and *B* causes *Y*_1_ to first increase and then decrease. When *A* is between 3.65 and 3.96 m^2^ and *B is* between 25.30 and 27.25 m/s, the response surface exhibits a peak, that is, *Y*_1_ reaches a maximum. **Figure 10.A.II** shows that for *B* = 27.59 m/s, increasing *A* and *C* causes *Y*_1_ to decrease: when *A* is below 3.65 m^2^, *Y*_1_ declines slowly with increasing *C*, whereas when *A* is above 3.65 m^2^, *Y*_1_ rapidly decreases with increasing *C*; at *C* = 1.8 m, increasing *A* causes *Y*_1_ to first increase to a maximum at *A* = 3.96 m^2^ and then decrease; at *C* = 2.7 m, *Y*_1_ gradually decreases with increasing *A*. **Figure 10.A.III** shows that at *B* = 21.96 m/s, *Y*_1_ decreases rapidly with increasing *C*, whereas at *B* = 33.21 m/s, *Y*_1_ remains nearly unchanged as *C* changes; at *C* = 1.8 m, *Y*_1_ decreases slowly with increasing *B*; and at *C* = 2.7 m, *Y*_1_ grows slowly with increasing *B*.

**Figure 10.B.I** shows that *Y*_2_ decreases noticeably with increasing *B* for small *A* values; with increasing *A*, *Y*_2_ increases up to *A* = 3.96 m^2^ and then decreases. **Figure 10.B.II** shows that *Y*_2_ decreases with increasing *C*. For large *A*, *Y*_2_ changes significantly with increasing *A*; that is, *Y*_2_ first increases slowly and then decreases. *Y*_2_ reaches a maximum at the smallest value of *C* and when *A* is within the range of 3.96-4.26 m^2^. **Figure 10.B.III** shows that *Y*_2_ decreases with increasing *B* and *C*, where *Y*_2_ changes significantly with *C*: these results are consistent with those presented in **Table 4**. This result shows that *C* has a significant impact on *Y*_2_.

**Figure 10.C.I** shows that *Y*_3_ declines with increasing *A* and *B*, where *Y*_3_ changes more significantly with increasing *B* than with increasing *A*, which indicates that changes in *B* impact *Y*_3_ more significantly than changes in *A*. **Figure 10.C.II** shows that with increasing *C*, *Y*_3_ decreases slowly up to *A* = 3.65 m^2^ and then rapidly decreases; with increasing *A*, *Y*_3_ increases slowly up to *C* = 2.25 m and then decreases. **Figure 10.C.III** shows that *Y*_3_ decreases with increasing *B* and *C* and reaches a maximum at *B* = 21.96 m/s and *C* = 1.80 m.

Combining the results of the analysis presented above with the variance analysis results presented in **Table 4** produces the following ranking (in order of decreasing significance) for the test factors: *C*, *A* and *B* for *Y*_1_ and *C*, *B* and *A* for *Y*_3_.

### 3.3 Orchard tests

The test data analysis method described in Section 2.6 was used to determine the quantities of spray deposited on different canopy regions for different test combinations. **Table 5** presents a comparison of these results with those calculated by the proposed computational model.

**Table 5:** Model verification test results.

Test No.	Inner deposition (mL·m^-2^)			Middle deposition (mL·m^-2^)			Outer deposition (mL·m^-2^)		
	Calculated value	Measured value	Error (%)	Calculated value	Measured value	Error (%)	Calculated value	Measured value	Error (%)
1	5.65	4.52	25.06	10.12	6.63	52.71	5.04	8.21	38.60
2	5.82	5.38	8.22	8.33	8.82	5.46	5.25	9.06	42.02
3	6.30	6.51	3.25	9.12	8.66	5.39	8.28	10.66	22.30
4	5.87	9.37	37.29	10.19	10.42	2.20	7.42	12.04	38.34
5	7.57	5.31	42.46	11.80	8.07	46.16	13.32	10.94	21.83

The model accuracy varies with the test combinations. As the spray distance decreases and the air speed increases, the calculation error of the model increases for the inner and middle regions of the canopy but decreases for the outer region of the canopy, and the calculation error of the model is relatively large when the air speed and spray distance are taken to the maximum or minimum value. The reason may be that there are some differences in contour between the artificial tree and the peach tree, and with the change of spray distance and air speed at the fan outlet, the deposition distribution of spray droplets in the vertical direction of the sprayer changes, resulting in differences in the spray deposited on the canopy of the at the fan outlet and the peach tree, which leads to the calculation error of the model. The calculated mean values of the relative error per unit area in the inner, middle and outer regions of the canopy are 23.26%, 22.38% and 32.62%, respectively.

## 4 Discussion

The calculation model for pesticide deposition on fruit tree canopies can be used to evaluate the efficacy of pesticide spraying in orchards and optimize sprayer parameters while providing data to help manage the tracing and quantification of pesticide application in orchards. Hong et al. (2018); Duga et al. (2015) and Zhu et al. (2022) studied the qualitative relationship between the operation parameters of orchard sprayers and the deposited pesticide distribution in canopies. However, the quantity of pesticide deposited on the canopy was not calculated. In this study, the key influencing factors for the deposited pesticide distribution in the canopy were used in an orthogonal test to establish a computational model for the quantity of pesticide deposited in different canopy regions.

The characteristics of fruit trees vary considerably with the tree type and growth period. To improve the application scope of the model, a reasonable range of test factors should be used the orthogonal regression modelling method, which is difficult to implement for real orchards. In this study, artificial trees were used to simulate real fruit frees. The canopy leaf area was manually changed to simulate changes in real fruit-tree canopies and thereby control the orthogonal test factors. A regression analysis was used to establish a computational model for the quantity of pesticide deposited on different canopy regions, and the model was verified using data for real fruit trees. The modelling method proposed in this paper can complement the existing CFD simulation modelling methods, which provides a novel insight for the construction of quantitative computational modelling of pesticide deposition on canopies using air-assisted orchard sprayers.

The computational model established using artificial trees can be applied to orchards. The maximum mean error of the model does not exceed 32.62%, showing that the model exhibits high accuracy for fruit tree canopies of different shapes. Recently, our research team made a breakthrough in LiDAR-based online computation of the fruit tree canopy leaf area (Gu et al., 2022). This method has considerable application value for determining the canopy leaf area of different types of fruit trees using LiDAR and can be used to investigate the universality of models and improve the calculation accuracy of models.

## 5 Conclusion

In this study, leaves were manually arranged to create artificial tree canopies with the following leaf areas: 2.54 m^2^, 3.05 m^2^, 3.81 m^2^, 4.57 m^2^ and 5.08 m^2^. An orchard air-assisted sprayer with airflow control was used to conduct pesticide application tests on artificial trees at different air speeds. Water-sensitive papers and filter papers used for sampling were placed at different positions in the canopies to determine the required range of the effective air speed as 18.12-37.05 m/s for canopy leaf areas ranging between 2.54 and 5.08 m^2^. The test factors included the canopy leaf area, air speed and airflow travel distance. A computational model for pesticide deposition in the inner, middle and outer regions of the canopy was established using a three-factor five-level quadratic general rotational orthogonal test; the *R* ^2^ values were 0.8199, 0.8575 and 0.9042, respectively. There is considerable variation among the characteristics of fruit tree canopies in real orchards. It is challenging to perform orthogonal regression modelling based on design parameters appropriate for real orchards. The computational model established in this study was developed using data for artificial trees, which provides novel concepts for formulating quantitative computational models for pesticide deposition on fruit tree canopies.

The significance of the influencing factors for pesticide deposition was analyzed based on a regression equation, regression coefficient and the lack-of-fit of the regression model. The results show that the influencing factors for the deposited pesticide distribution in the canopy can be ranked in decreasing order of significance as follows: the airflow travel distance, leaf area and air speed for the inner canopy region, followed by the airflow travel distance, air speed and leaf area for the middle and outer regions of the canopy. Tests were conducted on peach tree canopies to verify the model. The mean calculation errors of the computational model for pesticide deposition in the inner, middle and outer regions of the canopy were determined to be 23.26%, 22.38% and 32.62%, respectively. Studies will be conducted in the future to determine the canopy leaf areas of different types of fruit trees based on LiDAR, the universality of the model and ways to improve the calculation accuracy of the model.

## Data availability statement

The original contributions presented in the study are included in the article/supplementary material. Further inquiries can be directed to the corresponding authors.

## Author contributions

HD, ChaZ and ChuZ: conceptualization, validation, investigation and methodology. HD, SY and YG: software and visualization. HD, QL and SY: formal analysis. HD, ChaZ and KY: data curation. QL and ChuZ: resources and supervision. HD, QL and ChaZ: writing—original draft preparation. HD, ChaZ, SY, and YG: writing—review and editing and funding acquisition. ChaZ: project administration. All authors contributed to the article and approved the submitted version.
